# Breast may not always be best: moderation of effects of postnatal depression by breastfeeding and infant sex

**DOI:** 10.1186/s13293-021-00403-1

**Published:** 2021-11-07

**Authors:** Elizabeth C. Braithwaite, Helen Sharp, Andrew Pickles, Jonathan Hill, Nicola Wright

**Affiliations:** 1 Department of Psychology, Faculty of Health and Education, Manchester Metropolitan University, Brooks Building, 53 Bonsall Street, Manchester, M15 6GX UK; 2 Department of Primary Care and Mental Health, Faculty of Health and Life Sciences, University of Liverpool, Liverpool, UK; 3 Department of Biostatistics and Health Informatics, Institute of Psychiatry, Psychology and Neuroscience, Kings College London, London, UK; 4 School for Psychology and Clinical Language Sciences, University of Reading, Reading, UK

**Keywords:** Postnatal depression, Breastfeeding, Negative emotionality, Sex differences

## Abstract

**Background:**

There is good evidence that female infants are particularly vulnerable to poor emotional outcomes following in utero glucocorticoid exposure. It is currently unclear whether such effects might persist into the postnatal period for breastfed infants, as maternal cortisol is expressed in breastmilk and is influenced by maternal psychological distress. We pre-registered hypotheses that maternal postnatal depression would be associated with infant negative emotionality, and that this effect would be moderated by breastfeeding status and infant sex.

**Methods:**

We analysed data from the Wirral Child Health and Development Study (WCHADS), a prospective epidemiological study starting in pregnancy. Nine weeks after birth mothers self-reported depressive symptoms and breastfeeding status, and reported infant negative emotionality using the distress to limits subscale of the infant behaviour questionnaire (IBQ-R) when their infant was aged 9 weeks and 14 months. Maximum likelihood estimations made use of data from 857 mother-infant pairs.

**Results:**

At 9 weeks of age, maternal postnatal depressive symptoms were positively associated with infant distress to limits; however, this effect was not moderated by infant sex or breastfeeding. At age 14 months, the association between postnatal depression symptoms and distress to limits was greatest in the breastfed females, whereas the association was smaller, but still significant, in the non-breastfed females. For males, the association was non-significant in both the breastfed and non-breastfed groups. A test of sex difference between breastfed males and females was significant.

**Conclusions:**

We provide evidence that effects of maternal postnatal depression on child emotional outcomes are moderated by breastfeeding status and differ by infant sex. Female vulnerability to elevated maternal breastmilk glucocorticoids may, at least in part, explain these effects.

## Highlights

Female infants breastfed by mothers with high depressive symptoms showed the highest levels of negative emotionality at age 14 months.

Conversely, female infants breastfed by mothers with very low depressive symptoms showed the lowest negative emotionality.

Negative emotionality of males was unrelated to maternal postnatal depression and breastfeeding at 14 months of age.

We provide novel evidence that effects of maternal postnatal depression on infant emotional outcomes is moderated by breastfeeding status and differs by infant sex.

## Background

Fetal programming is the adaptive process by which maternal cues about the environment impact on fetal development in utero, to improve offspring fitness for the postnatal environment [1]. Although this theory was first proposed to account for associations between low birthweight and later obesity, cardiovascular disease and diabetes [2], it has been applied to explain the well-replicated finding that maternal prenatal psychological stress increases risk for poor offspring outcomes [3, 4]. The prevailing mechanistic theory to account for mood-associated effects on offspring outcomes is via changes in the maternal and fetal hypothalamic–pituitary adrenal (HPA) axes [3, 4]. Low-mood associated increases in maternal glucocorticoids are actively transferred to the fetal blood circulation [5], which perturb the development of the fetal HPA axis [6–8]. Such changes to the fetal/infant HPA axis confer susceptibility to later psychopathology [8], and accumulating evidence suggests that these effects are sex-dependent. For example, exposure to prenatal psychological stress/increased maternal cortisol is associated with reduced birthweight [9], fearful temperament [10], increased negative emotionality [11, 12], changes in HPA function [8], adolescent depression [8], and changes in amygdala size [13] and connectivity [14, 15], all in females only. This suggests that females are specifically susceptible to maternal psychological distress in early development, mediated by glucocorticoid pathways.

It is unclear, however, whether such susceptibility continues beyond gestation for breastfed infants, since maternal glucocorticoids are expressed in breastmilk, and their levels are influenced by maternal psychological distress [16]. There is some supportive evidence from a study of rhesus macaques; a high level of maternal glucocorticoids expressed in breastmilk predicted a more nervous and less confident temperament in both male and female offspring [17]. In human research, a higher level of plasma cortisol has been associated with infant fear at 2 months of age among breast-fed, but not formula-fed, infants [18], suggesting that effects were mediated via breastmilk glucocorticoids. In addition, a direct relationship between breast milk cortisol and negative affect has been demonstrated in a small sample of 53 3-month-old infants, an effect that was only evident in females [19]. It is of course possible that other mechanisms may mediate these effects, such as the impact of breastfeeding on the seeding of the infant’s microbiome [20], and the resultant role of the microbiome in mental health over the life course [21].

We have previously shown that maternal postnatal depression is positively associated with infant distress to limits [11] and negative emotionality [12] in females, but not males. The current research aims to examine whether such effects are moderated by breastfeeding status, informed by the hypothesis that those infants who are breastfed by depressed mothers may be exposed to greater levels of maternal glucocorticoids. Unfortunately, in the present study we were unable to measure breastmilk glucocorticoids directly; therefore, we use breastfeeding status (exclusively breastfeeding vs not) as a proxy for breastmilk glucocorticoid exposure. In addition, we aim to test for effects by sex, informed by the emerging literature, which indicates that female infants are susceptible to early-life stress, mediated by glucocorticoid mechanisms.

### Hypotheses

1. Maternal postnatal depressive symptoms will be positively associated with infant distress to limits at ages 9 weeks and 14 months, and the strength of the association will be greater for infants who are breastfed compared with those who are not.
2. Moderation of the effect of postnatal depressive symptoms on infant distress to limits by breastfeeding will be evident for females but not males.

## Methods

### Design

Participants were members of the Wirral Child Health and Development Study (WCHADS), a prospective, epidemiological study starting in pregnancy [22]. Mothers provided informed consent at the initial recruitment at the antenatal clinic. All procedures contributing to this work were approved by the Cheshire North and West Research Ethics Committee.

### Sample

The sample comprises 1233 women recruited in pregnancy with a live, singleton baby for long-term follow-up post-birth. The mean age at recruitment was 26.8 years (SD = 5.8, rage = 18–51), 41.8% of the sample were in the most deprived quintile of UK neighbourhoods [23] and 96.1% indicated that they were White British. The analyses presented here utilise data collected at 9 weeks after birth, and when the infants were 14 months. Mothers self-reported symptoms of postnatal depression when their infants were 9 weeks and also reported on breastfeeding status, and feeding behaviours, at this time. In addition, mothers reported on infant behaviours when their infant was aged 9 weeks, and again at 14 months of age. A total of 857 mothers provided breastfeeding and postnatal depression data at age 9 weeks and form the sample analysed here. Of these, 656 provided age 9 weeks IBQ-R data and 683 provided age 14 month IBQ-R data, but maximum likelihood estimation was used to analyse data from the full sample of 857.

### Measures

Postnatal depression. Maternal postnatal depression was assessed at 9 weeks after birth using the Edinburgh Postnatal Depression Score (EPDS), the most widely used self-report questionnaire to identify symptoms of depression during the perinatal period [24]. The scale consists of ten items that describe common symptoms of depression. Each item is scored from 0 to 3, and the scale has a maximum of 30, with a score of 13 or above indicative of clinically significant depression. The total score was used for analysis.

Breastfeeding status. Mothers reported on feeding behaviours 9 weeks after birth. They were asked ‘When your baby had reached 6 weeks old, did you…’ and were required to respond on a 7-point Likert-type scale from ‘Mostly breastfed’ to ‘Mostly bottle fed’. They were also asked to respond to the question ‘When your baby had reached 6 weeks old, did you bottle feed using formula milk or breast milk or both?’, and were required to choose one of the following responses: formula milk, breast milk, breast and formula milk, do not bottle feed. Mothers who indicated that they were exclusively breastfeeding, or giving their baby exclusively breast milk via a bottle, were grouped into the ‘breastfeeding group’, all remaining mothers were grouped into the ‘non-breastfeeding group’. This dichotomous grouping was pre-specified in the pre-registration.

Infant distress to limits. At 9 weeks and 14 months of age, mothers reported their infant’s level of behavioural reactivity using the distress to limitations subscale of the Infant Behaviour Questionnaire—Revised (IBQ-R) [25]. Mothers were required to report how often their infant engaged in various behaviours that reflect fussing, crying or showing frustration, during the past week using a 7-point Likert scale from ‘Never’ to ‘Always’. The IBQ-R has established reliability and validity, has been widely used in developmental studies [26, 27], and we have previously used this measure with 8-week-old infants [11].

Confounders. We took account of the following confounders, for which occasional missing values (less than 1%) have been previously imputed, because of their established association with child behaviour development: maternal age and education, marital status, smoking in pregnancy, postcode based neighbourhood deprivation [23], infant birthweight by gestational age and obstetric risk score from hospital records. We also include mothers self-report of infant stroking at age 9 weeks as a confound for the touch element of breastfeeding [28].

### Statistical analysis

The analysis plan (https://osf.io/yezn5/?view_only=4dc2f0b63af8411cbaad62e8aba566cf) and hypotheses were pre-registered. Analysis was conducted in Stata version 14 using the sem command to conduct path analysis using the full-information maximum-likelihood mlmv estimator. Following square root transformations for skewed variables (EPDS and 9 week and 14 month IBQ-R) bivariate associations between the study variables were assessed using Pearson, point-biserial and tetrachoric correlations. Separate models predicting age 9 week IBQ-R and age 14 month IBQ-R were examined. In the first model, the confounding variables, main effects of postnatal depression symptoms (continuous variable) and breastfeeding status (binary variable) and the interaction term between postnatal depression and breastfeeding status was entered. As we a priori hypothesised that the association between postnatal depression and infant IBQ-R will be stronger in breastfed infants, we then estimated models testing the main effect of postnatal depression in the breastfed and non-breastfed groups. As the moderation of postnatal depression by breastfeeding is hypothesised to be present in females only, all models were estimated separately in males and females, and a Wald test for a sex difference in the postnatal depression and IBQ-R association performed.

## Results

The descriptive statistics and bivariate associations for the key study variables are presented in Tables 1 and 2, for males and females separately. The IBQ-R distress to limits subscale showed small to moderate associations from 9 weeks to 14 months in both males (*r* = 0.21) and females (*r* = 0.28). At age 9 weeks, maternal depression showed a small to moderate positive correlation with concurrently assessed IBQ-R distress to limits in both males and females but showed an association to age 14 month IBQ-R distress to limits only in females. The confounding variables were largely not associated with maternal depression or infant IBQ distress to limits, but not engaging in breastfeeding at 6 weeks after birth was associated with lower maternal age, being single, leaving education at age 16 years, belonging to the most deprived quintile of UK neighbourhoods and smoking during pregnancy.

**Table 1:** Bivariate associations and descriptive statistics for males (*N* = 433)

	9 week IBQ	14 month IBQ	9 week EPDS	Breast fed	Younger maternal age	Stroking	Birthweight by gestational age	Obstetric risk	Left school aged 16	Not married	Smoked	Most deprived quintile
9 week IBQ		0.21***	0.26***	0.07	− 0.05	0.01	− 0.07	0.03	− 0.04	0.05	− 0.01	− 0.07
14 month IBQ			0.04	− 0.03	0.08	0.08	0.02	0.06	0.01	0.11^+^	0.07	0.02
9 week EPDS				0.04	0.02	0.01	− 0.02	− 0.07	0.03	0.12*	0.09^+^	0.04
Breastfed					− 0.14**	0.02	0.01	− 0.02	− 0.37***	− 0.20^+^	− 0.43***	− 0.24**
Mean	2.60	3.43	5.87		**28.64**	3.88	87.10	2.21				
SD	0.63	0.94	4.86		5.50	0.74	12.39	1.21				
*N*				112					89	77	48	145
%				25.6					21.0	78.2	11.5	34.2

Significant results are highlighted in bold

*IBQ* Infant Behaviour Questionnaire, *EPDS* Edinburgh Postnatal Depression Scale

*p < 0.05, **p < 0.01, ***p < 0.001

**Table 2:** Bivariate associations and descriptive statistics for females (*N* = 424)

	9 week IBQ	14 month IBQ	9 week EPDS	Breast fed	Younger maternal age	Stroking	Birthweight by gestational age	Obstetric risk	Left school aged 16	Married	Smoked	Most deprived quintile
9 week IBQ		0.28***	0.31***	0.08	− 0.05	0.01	− 0.02	− 0.04	− 0.04	0.01	− 0.11^+^	− 0.06
14 month IBQ			0.20***	− 0.04	0.17**	− 0.03	− 0.05	0.06	0.04	0.11*	− 0.01	0.01
9 week EPDS				− 0.08	0.05	0.01	− 0.02	− 0.03	− 0.01	0.10*	− 0.01	0.01
Breastfed					− 0.15***	− 0.02	0.05	0.09^+^	− 0.37***	− 0.41***	− 0.43***	− 0.28**
Mean	2.53	3.26	5.41		26.62	3.86	84.41	2.20				
SD	0.60	0.97	4.61		5.39	0.72	12.20	1.20				
*N*				105					99	75	50	131
%				25.1					22.9	17.3	11.7	37.4

Significant results are highlighted in bold

*IBQ* Infant Behaviour Questionnaire, *EPDS* Edinburgh Postnatal Depression Scale

*p < 0.05, **p < 0.01, ***p < 0.001

Path analysis using the sem command in Stata was used to test the study hypotheses. Separate models were run for age 9 week and age 14 month outcomes, and all models were run as multi-group by child sex. All models included the identified socio-demographic, obstetric and parental touch confounding variables (coefficients not shown). In initial models we tested for an interaction between breastfeeding status and age 9 week EPDS and IBQ-R distress to limits outcome. For age 9 week IBQ-R distress to limits, the interaction term between maternal postnatal depression and breastfeeding was non-significant in both males (standardized coeff = 0.16 (95% CI − 0.10 to 0.42), *p* = 0.221) and females (standardized coeff = − 0.03 (95% CI − 0.24 to 0.19) *p* = 0.805). The main effect of depression was significant for both males and females (standardized coeff = 0.29 (95% CI 0.18 to 0.39), *p* < 0.001; and standardized coeff = 0.33 (95% CI 0.23 to 0.42), *p* =  < 0.001), respectively), whereas breastfeeding showed no significant main effect. For 14 month IBQ-R, the interaction term was non-significant for males (standardized coeff = − 0.13 (95% CI − 0.39 to 0.13) *p* = 0.331) but for females, consistent with hypotheses, the interaction approached significance (standardized coeff = 0.19 (95% CI − 0.01 to 0.39) *p* = 0.063). Results are displayed in Table 3.

**Table 3:** Results of sem models testing the interaction term between breastfeeding and depression symptoms predicting age 9 week and 14 month IBQ distress to limits, after accounting for confounding variables

*N* = 857	Males			Females		
	Unstand. coeff (95% CI)	Stand. coeff (95% CI)	*p*	Unstand. coeff (95% CI)	Stand. coeff (95% CI)	*p*
Age 9 week IBQ distress to limits						
Age 9 week maternal depression	**0.05 (0.03 to 0.07)**	**0.29 (0.18 to 0.39)**	** < 0.001**	**0.06 (0.04 to 0.07)**	**0.33 (0.23 to 0.42)**	** < 0.001**
Breastfeeding status	0.04 (− 0.07 to 0.15)	0.02 (− 0.03 to 0.06)	0.463	0.04 (− 0.01 to 0.09)	0.10 (− 0.01 to 0.20)	0.068
Depression × breastfeeding	0.03 (− 0.02 to 0.07)	0.16 (− 0.10 to 0.42)	0.221	− 0.01 (− 0.05 to 0.03)	− 0.03 (− 0.24 to 0.19)	0.805
Age 14 month IBQ distress to limits						
Age 9 week maternal depression	0.02 (− 0.09 to 0.19)	0.07 (− 0.05 to 0.20)	0.261	**0.03 (0.01 to** **0.06)**	**0.14 (0.02 to 0.25)**	**0.024**
Breastfeeding status	0.05 (− 0.09 to 0.19)	0.09 (− 0.16 to 0.33)	0.489	− 0.11 (− 0.23 to 0.02)	− 0.17 (− 0.37 to 0.03)	0.097
Depression × breastfeeding	− 0.03 (− 0.09 to 0.03)	− 0.13 (− 0.39 to 0.13)	0.311	0.05 (− 0.01 to 0.11)	0.19 (− 0.01 to 0.39)	0.063

Significant results are highlighted in bold

**Fig. 1:**
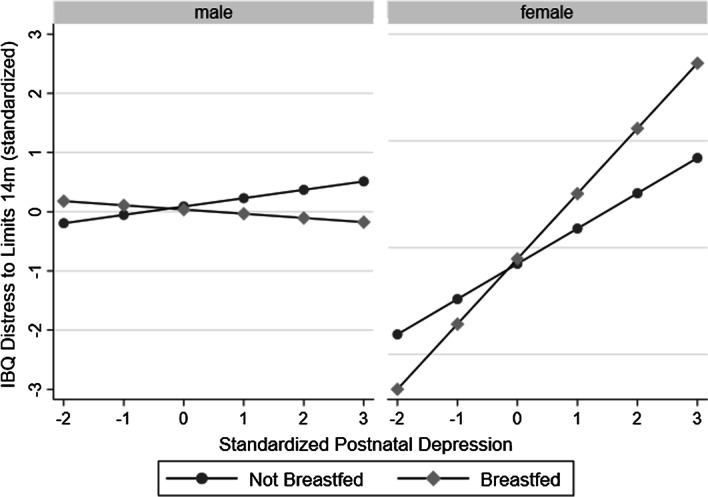
Plot showing the association between postnatal depression symptoms and age 14 month IBQ-R in breastfed and non-breastfed males and females (after accounting for confounders)

As pre-specified, we then estimated further models examining the association between postnatal depression symptoms and IBQ-R distress to limits at 9 weeks and 14 months in males and females who were breastfed and not breastfed. At age 9 weeks, and consistent with the non-significant interaction terms, the association between depression and concurrently assessed IBQ-R was very similar across the four groups. At age 14 months, consistent with hypotheses, the association between higher depression scores and higher IBQ-R distress to limits was largest in the breastfed females. The association was significant but smaller in the non-breastfed females. For males the association was non-significant in the non-breastfed and breastfed groups. The Wald test of a sex difference in the association between postnatal depression and IBQ-R was significant (chi2(1) = 3.40, *p* = 0.024). Results are displayed in Table 4 and graphically in Fig. 1.

**Table 4:** Results of models testing the association between postnatal depression and IBQ-R at 9 weeks and 14 months in males and females who were breastfed and not breastfed

	Males						Females					
	Breastfed			Non-breastfed			Breastfed			Non-breastfed		
	Unstand. coeff (95% CI)	Stand. coeff (95% CI)	*p*	Unstand. coeff (95% CI)	Stand. coeff (95% CI)	*p*	Unstand. coeff (95% CI)	Stand. coeff (95% CI)	*p*	Unstand. coeff (95% CI)	Stand. coeff (95% CI)	*p*
Age 9 week IBQ distress to limits												
Age 9 week maternal depression	**0.07** **(0.03 to 0.11)**	**0.38** **(0.18 to 0.57)**	** < 0.001**	**0.04 (0.02 to 0.07)**	**0.26 (0.13 to 0.38)**	** < 0.001**	**0.05** **(0.02 to 0.09)**	**0.31** **(0.10 to 0.51)**	**0.006**	**0.06** **(0.04 to 0.07)**	**0.34 (0.23 to 0.44)**	** < 0.001**
Age 14 month IBQ distress to limits												
Age 9 week maternal depression	− 0.01 (− 0.07 to 0.05)	− 0.03 (− 0.25 to 0.19)	0.784	0.02 (− 0.01 to 0.05)	0.08 (− 0.05 to 0.21)	0.253	**0.08** **(0.04 to 0.13)**	**0.34** **(0.15 to 52)**	**0.001**	**0.03** **(0.01 to 0.06)**	**0.13 (0.01 to 0.25)**	**0.030**

Significant results are highlighted in bold

## Discussion

We pre-registered the hypothesis that maternal postnatal depression symptom level would be positively associated with infant distress to limits at ages 9 weeks and 14 months, and the strength of the association would be greater for infants who were breastfed compared with those who were not. In addition, we hypothesised that this effect would be present in female, but not male, infants. Our data analysis partly supported these hypotheses. We provide evidence that associations between maternal postnatal depression symptoms at 9 weeks after birth, and infant distress to limits at 14 months of age, are moderated by breastfeeding status and differ by infant sex. That is, females who were breastfed by mothers with a higher level of depressive symptoms showed the greatest distress to limits at 14 months of age, and conversely, females breastfed by mothers with lower symptoms of depression showed the lowest levels of distress to limits. For males, there was no association between maternal postnatal depression symptoms and distress to limits at 14 months of age in neither the breastfed nor the non-breast-fed groups, as hypothesised.

This finding is consistent with analyses from a much smaller sample of 53 infants, which directly measured breastmilk cortisol, and demonstrated that it was positively associated with negative affect, but only among female infants [19]. Although we did not directly measure breastmilk cortisol, we used a dichotomous measure of breastfeeding in an attempt to index breastmilk cortisol exposure. Our findings are also consistent with a wealth of emerging research from the prenatal period, which suggests that females are particularly vulnerable to the adverse effects of maternal stress and depression during pregnancy [3, 4, 8, 10–12]. The theory is that elevated levels of maternal cortisol cross the placental barrier, and in response the size and connectivity of the amygdala is altered in females [13–15], which increases risk for later emotional dysregulation and affective outcomes. Here we provide evidence that vulnerability to glucocorticoid-mediated risk may continue into the postnatal period for those breastfed females whose mothers experience symptoms of postnatal depression. However, we acknowledge that our index of breastmilk cortisol exposure, based on maternal reports of breastfeeding status, is an imperfect approach, and further research is needed, where glucocorticoids in breastmilk are directly measured, and longitudinal associations with child emotional outcomes are tested.

We did not, however, find supporting evidence for our hypotheses in cross section at 9 weeks after birth. At this time point, there was a positive association between maternal postnatal depression symptoms and infant distress to limits in both breastfed and non-breastfed infants, and both males and females. However, there was no moderation by breastfeeding status in neither male nor female groups. One possibility is that the level and variability of distress to limitations reported using the IBQ-R increase with infant age [25], which we also see here (see means and standard deviations in Table 1). Therefore, reduced variability could have reduced the power to detect moderating effects of breastfeeding. Shared method variance could also have influenced the findings, as mothers were reporting on their own depressive symptoms and their child’s negative emotionality at the same time point. The hypotheses made in this paper are based on the idea that early exposure to maternal glucocorticoids can influence emotional development in children. However, the full extent to which glucocorticoids can influence early neurodevelopment is currently unknown. Therefore, it could be that just 9 weeks exposure to elevated glucocorticoids in breastmilk is insufficient for effects on negative emotionality in children to become apparent. Unfortunately, we were unable to test whether the duration of breastfeeding was important for child outcomes in this study because that information was not reported by mothers; however, this is certainly an important question for future research.

An important consideration when appraising the results presented here is that breastmilk cortisol might not be the mechanism that mediates associations between maternal postnatal depression and negative emotionality in females, and in fact, this could be a proxy for something else. For example, there is evidence that aspects of the maternal microbiome are transferred via breastmilk [29, 30], and that early seeding of the human microbiome in infancy is moderated by both sex and feeding type [20]. It is also evident that there are sex differences in the microbiome throughout the life course [31] and we know that the microbiome plays an important role in mental health and wellbeing [21], which also appears to be sex-dependant [32, 33]. Thus, our findings presented here could, at least in part, be explained by sex-specific seeding of the microbiome during early life, which is moderated by breastfeeding status. In addition, there are also sex-specific changes in breastmilk composition across the early life course [34], including changes in nutrient composition and immune factors; however, it is currently unknown whether there are sex-specific nutritional and immunological changes in breastmilk in relation to maternal postnatal depression. An alternative explanation for the current findings is that mothers with depressive symptoms may rate breastfed females differently from breastfed males; however, there is no existing literature to support this hypothesis.

Strengths of the current study include prospective, longitudinal assessments of both mother and child using validated measures. In addition, our analyses accounted for many potentially confounding variables, including maternal-reported rates of infant stroking, to account for the possible effects of increased touch associated with breastfeeding mediating the postnatal depression negative emotionality association. We also accounted for several variables in the postnatal environment which were associated with maternal breastfeeding status, including: maternal age, education, marital status, deprivation, and smoking. The postnatal environment as a whole is an important consideration in the associations between breastfeeding and child outcomes, and including variables which are related to breastfeeding status in our analyses improves confidence in the findings presented here. The hypotheses tested here were theory-driven based on our previous work, and pre-registered, which is another key strength. The main limitation of this study is that ratings of child negative emotionality were based purely on maternal report and, therefore, may be subject to reporter bias, though we have no reason to expect that effects of reporter-bias may be sex-specific. A future line of investigation will be to attempt to replicate these analyses using partner-reports of child negative emotionality, and also using teacher-reports at a later time point. It would also be of great interest and significance for future research to directly test whether breastmilk cortisol mediates associations between maternal postnatal depression and infant negative emotionality in a sex-specific manner, as well as other nutritional and immunological components.

## Conclusion

In sum, we developed, pre-registered and tested hypotheses based on evidence from the prenatal programming literature. Our rationale was that if effects of maternal prenatal stress on child development were mediated by maternal glucocorticoids, and that females are particularly vulnerable to poor emotional outcomes in this context, then such effects might continue into the postnatal period for those infants who are breastfed because maternal glucocorticoids are present in breastmilk. This is the first study to provide evidence that breastfeeding moderates the effects of maternal postnatal depression, and that the effects differ by sex, which has implications for both clinical practice and public health messaging. The direct effects of breastmilk cortisol on child development are currently unclear; however, the findings presented here provide clear impetus for replication and further investigation.

## Data Availability

Due to ethical constraints supporting data cannot be made openly available. Supporting data are available to bona fide researchers on approval of an application for access. Further information about the data and conditions for access are available at the University of Liverpool Research Data Catalogue: 10.17638/datacat.liverpool.ac.uk/564.
